# Acute Cholangitis With Non-O1 and Non-O139 Vibrio cholerae Bacteremia: A Case Report

**DOI:** 10.7759/cureus.89160

**Published:** 2025-07-31

**Authors:** Yoshinori Takahashi, Ryosuke Isshiki, Hajime Sunagozaka, Eiji Arakawa, Shigehiko Karino

**Affiliations:** 1 Department of General Medicine and Infectious Diseases, Japan Agricultural Cooperative (JA) Toyama Koseiren Takaoka Hospital, Takaoka, JPN; 2 Department of Gastroenterology, Japan Agricultural Cooperative (JA) Toyama Koseiren Takaoka Hospital, Takaoka, JPN; 3 Department of Bacteriology, Japan Institute for Health Security, National Institute of Infectious Diseases, Shinjuku, JPN

**Keywords:** bacteremia, cholangitis, gastroenteritis, novc, vibrio cholerae

## Abstract

We describe a case of acute cholangitis with bacteremia caused by non-O1 and non-O139 *Vibrio cholerae* (NOVC) in a 76-year-old man following acute gastroenteritis. The strain, isolated from blood cultures, was identified as serogroup O120. It lacked the cholera toxin gene, but it harbored multiple virulence genes, including *hlyA*, *rtxA*, *vscV2*, and *vspD*. Risk factors included total gastrectomy, diabetes, and prior biliary interventions. Although the patient’s diarrhea had resolved by the time of admission, dietary history and timely blood cultures enabled an accurate diagnosis. This case highlights the invasive potential of NOVC strains harboring alternative virulence determinants in immunocompromised hosts.

## Introduction

Cholera is a severe diarrheal disease caused by *Vibrio cholerae* [1]. *V. cholerae* is motile, comma-shaped, facultative anaerobic, Gram-negative aquatic bacterium that is widely distributed in the marine environment, particularly in estuaries, and is a major cause of fatal infections in many developing regions where clean drinking water is not readily available [1]. *V. cholerae* is classified into more than 200 serotypes based on the O antigen of its lipopolysaccharide. Among these, the O1 and O139 serogroups, which produce cholera toxin, are responsible for epidemic cholera [1]. In contrast, most strains of non-O1 and non-O139 *V. cholerae* (NOVC) do not possess cholera toxin and either persist asymptomatically or cause sporadic diseases such as self-limiting gastroenteritis in a healthy host. The occurrence of disease depends on the specific combination of virulence genes harbored by the strain and the hosts’ health status [2,3]. In recent years, reports of infections caused by NOVC have been increasing, including a growing number of extraintestinal infections such as wound infections, bacteremia, urinary tract infections, and meningitis [2,4]. This can be fatal, especially in immunocompromised patients [2,3]. Herein, we describe a case of acute cholangitis with NOVC bacteremia that occurred following acute gastroenteritis.

## Case presentation

The patient was a 76-year-old Japanese man. In August, during the summer season, he developed vomiting and diarrhea that began seven days prior to admission, followed by fever with shaking chills two days before presentation. He was transported to our hospital for further evaluation. Contrast-enhanced computed tomography revealed no abnormalities suggestive of gastroenteritis and biliary tree infection, such as thickened gastrointestinal wall, biliary stones, bile duct dilatation, and uneven contrast effect in liver parenchyma; however, he exhibited right upper quadrant tenderness, a positive liver percussion sign, and elevated hepatobiliary enzymes, leading to a clinical diagnosis of acute cholangitis and subsequent hospitalization. At the time of admission, his diarrhea had resolved; therefore, stool culture was not obtained. After two sets of blood cultures were drawn, empirical treatment with cefmetazole (1 g every 8 h) was initiated. His past medical history included total gastrectomy for gastric cancer with Roux-en-Y reconstruction, endoscopic sphincterotomy and stone removal for choledocholithiasis, cholecystectomy for cholelithiasis, hypertension, and type 2 diabetes mellitus. The patient had received prescriptions for amlodipine and lansoprazole; however, he did not receive any oral hypoglycemic agents or insulin. He had no history of allergies or smoking and drank alcohol only occasionally. Given his preceding gastrointestinal symptoms, a detailed dietary history was obtained, which revealed he had been frequently consuming sashimi prepared from juvenile yellowtail (*Seriola quinqueradiata*) purchased at a local grocery store. His cohabiting wife, who had no underlying conditions, had eaten the same food but remained asymptomatic.

Upon admission, he was alert and oriented, with the following vital signs: body temperature 38.2°C, blood pressure 122/78 mmHg, heart rate 90/min, respiratory rate 20/min, and saturation of percutaneous oxygen 97% on room air. Physical examination revealed no conjunctival icterus and no abnormal lung sounds or cardiac murmurs. Tenderness in the right upper quadrant and a liver percussion sign were positive. Laboratory tests showed a white blood cell count of 18,200/μL (neutrophils 86.5%), hemoglobin 13.1 g/dL, and platelet count 16.0 ×10^4^/μL. Liver function tests revealed abnormalities, with aspartate aminotransferase 125 U/L, lactate dehydrogenase 409 U/L, gamma-glutamyl transferase 94 U/L, and total bilirubin 1.9 mg/dL, suggestive of relatively hepatocellular-dominant liver disorder, but renal function was within normal range. Electrolyte abnormalities were noted: sodium 132 mEq/L and potassium 2.7 mEq/L. Plasma glucose level was 220 mg/dL, and an elevated hemoglobin A1c (HbA1c) level of 9.6% was noted, indicating poor glycemic control (Table 1). Abdominal contrast-enhanced computed tomography imaging revealed no biliary stones or bile duct dilatation suggestive of biliary obstruction or stricture and intestinal wall thickening suggestive of enteritis.

**Table 1:** Laboratory data upon the day of admission to our hospital. ALB: albumin, ALP: alkaline phosphatase, ALT: alanine aminotransferase, AST: aspartate aminotransferase, BUN: blood urea nitrogen, Cl: chloride, Cr: creatinine, CRP: C-reactive protein, D-Bil: direct bilirubin, γ-GTP: gamma-glutamyltransferase, Hb: hemoglobin, Ht: hematocrit, K: potassium, LDH: lactate dehydrogenase, Na: sodium, PG: plasma glucose, PLT: platelets, PT: prothrombin time, PT-INR: prothrombin time-international normalized ratio, RBC: red blood cell, T-Bil: total bilirubin, TP: total protein, WBC: white blood cell.

	Value	Normal range		Value	Normal range
Blood count			AST (U/L)	125	13-30
WBC (×10^3^/μL)	18.2	3.30-8.60	ALT (U/L)	59	10-42
Neutrophils (%)	86.5	38.5-80.5	LDH (U/L)	409	124-222
RBC (×10^6^/μL)	4.51	4.35-5.55	ALP (U/L)	177	38-113
Hb (g/dL)	13.1	13.7-16.8	γ-GTP (U/L)	94	13-64
Ht (%)	37.1	40.7-50.1	T-Bil (mg/dL)	1.9	0.4-1.5
PLT (×10^4^/μL)	16.0	15.8-34.8	D-Bil (mg/dL)	0.9	0.0-0.4
Serum chemistry			TP (g/dL)	5.9	6.6-8.1
BUN (mg/dL)	12.9	8.0-20.0	ALB (g/dL)	2.6	4.1-5.1
Cr (mg/dL)	0.88	0.65-1.07	CRP (mg/dL)	22.7	0.0-0.1
Na (mEq/L)	132	138-145	Coagulation		
K (mEq/L)	2.7	3.6-4.8	PT (%)	151	70-130
Cl (mEq/L)	95	101-108	PT-INR	0.81	0.8-1.2

**Figure 1:**
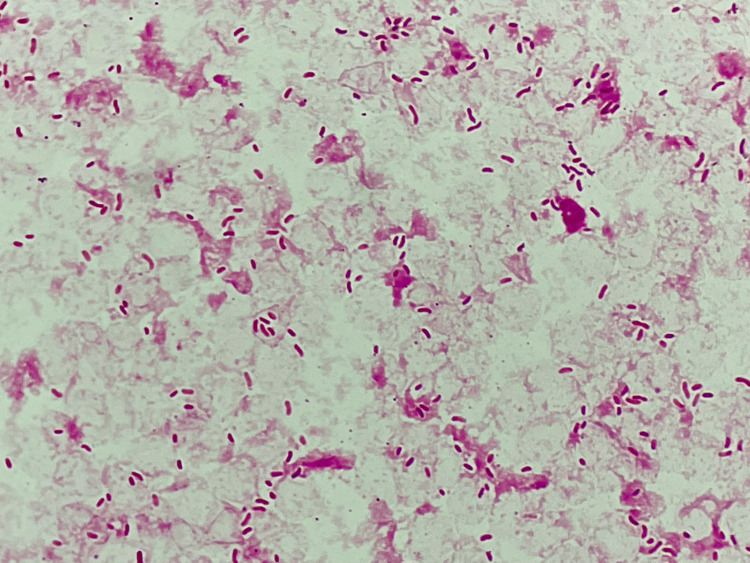
Gram staining of blood cultures revealed the presence of comma-shaped Gram-negative rod bacteria (×1000).

**Figure 2:**
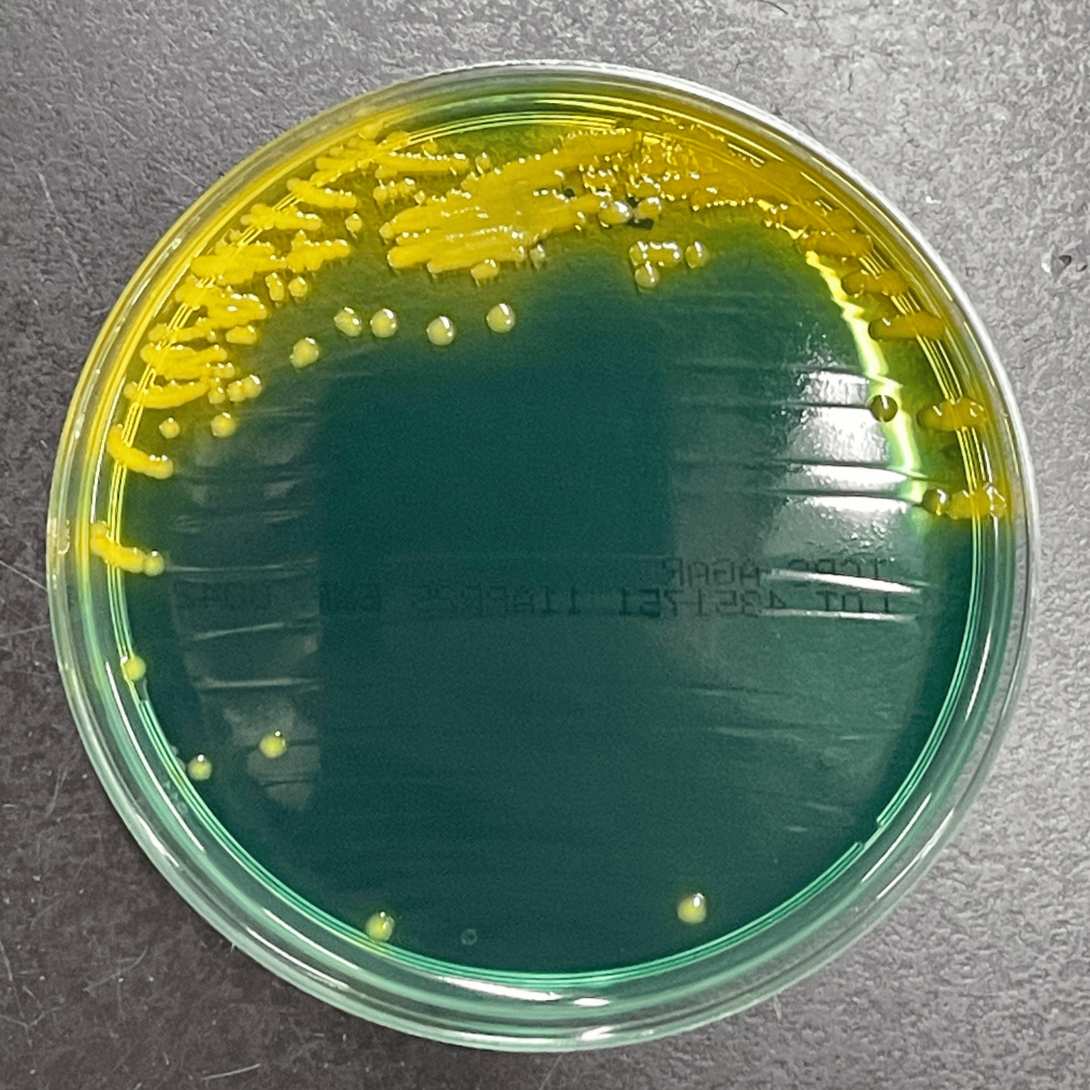
Yellow colonies of non-O1 and non-O139 Vibrio cholerae (NOVC) in the present case grown on thiosulfate-citrate-bile salts-sucrose agar.

On the second hospital day, Gram-negative rods were isolated from both sets of blood cultures and found to be slightly curved in shape (i.e., comma-shaped) (Figure 1). Given the summer season and dietary history of raw seafood consumption, *Vibrio* species were suspected. Empiric antibiotics were changed to ceftriaxone (2g every 24 h) based on previous case series of *Vibrio* species bacteremia (mainly non-O1 and non-O139 *Vibrio cholerae* (NOVC) or *V. vulnificus*) [5,6], and metronidazole (500 mg every 12 h) for covering anaerobic bacteria such as *Bacteroides fragilis*. Due to the absence of sepsis, the decision was made against administering antibiotic combination therapy for *Vibrio* species. The organism grew well on standard media the next day, including sheep blood agar, chocolate agar, and bromothymol blue agar. Matrix-assisted laser desorption ionization time-of-flight mass spectrometry identified *V. cholerae* with a score of 2.2. Subsequently, yellow colonies were observed on thiosulfate-citrate-bile salts-sucrose agar (Figure 2), and biochemical testing was positive for lysine decarboxylase, arginine dihydrolase, ornithine decarboxylase, arabinose utilization, and showed growth inhibition in 8% sodium chloride, consistent with *V. cholerae*. Serotyping for O1 and O139 antigens was negative, confirming the organisms as NOVC. Antimicrobial susceptibility testing was performed in accordance with Clinical and Laboratory Standards Institute guideline M45 [7]. The isolate was susceptible to all tested agents except imipenem, for which the minimum inhibitory concentration was 2 μg/mL (intermediate) (Table 2). Due to limited data on optimal treatment regimens for NOVC bacteremia, further de-escalation was not pursued. Since no evidence of biliary obstruction was observed, biliary drainage was not performed. The patient promptly became afebrile, and liver enzyme levels improved, indicating a favorable clinical course. Antibiotic therapy was completed in 14 days, and the patient was discharged. The local public health authority was notified, but no foodborne illness outbreak was reported.

**Table 2:** Antimicrobial susceptibility test for the strain of non-O1 and non-O139 Vibrio cholerae in the present case. ABPC: ampicillin, AMK: amikacin, AZT: aztreonam, CAZ: ceftazidime, CCL: cefaclor, CEZ: cefazolin, CFPM: cefepime, CFPN: cefcapene, CMZ: cefmetazole, CPFX: ciprofloxacin, CPZ: cefoperazone, CTM: cefotiam, CTRX: ceftriaxone, CTX: cefotaxime, FMOX: flomoxef, FOM: fosfomycin, GM: gentamicin, I: intermediate, IPM: imipenem, MEPM: meropenem, MIC: minimum inhibitory concentration, MINO: minocycline, N/A: not applicable, PIPC: piperacillin, S: susceptible, SBT: sulbactam, ST: sulfamethoxazole-trimethoprim, TAZ: tazobactam. * Cefazolin demonstrates susceptibility if the MIC is two or less, and intermediate susceptibility if the MIC is four. However, the panel utilized in this study lacked the capacity to measure MICs below four, consequently precluding the determination of applicable interpretation categories. # The measurement of ciprofloxacin was executed through the utilization of the disk diffusion method.

Antibiotics	MIC (μg/mL)	Interpretation category	Antibiotics	MIC (μg/mL)	Interpretation category
ABPC	≤8	S	CCL	≤8	−
PIPC	≤8	S	CFPN	≤0.25	−
ABPC/SBT	≤8	S	FMOX	≤8	−
PIPC/TAZ	≤16	S	IPM	2	I
CEZ*	≤4	N/A	MEPM	≤1	S
CTM	≤8	−	AZT	≤4	−
CAZ	≤4	S	AMK	≤4	S
CTX	≤1	−	GM	≤2	S
CTRX	≤1	−	MINO	≤2	−
CFPM	≤2	S	CPFX#	−	S
CPZ/SBT	≤16	−	FOM	16	−
CMZ	≤8	−	ST	≤2	S

Subsequently, further microbiological analysis was performed on this strain and identified the O serogroup as O120. Polymerase chain reaction analysis for virulence genes revealed that the strain was negative for the cholera toxin gene (*ctx*) and the heat-stable enterotoxin gene (*nag-st*) but positive for the El-Tor variant of hemolysin (*hlyA*), type III secretion system genes (*vscV2* and *vspD*), and repeat-in-toxin (*rtxA*). These analyses were performed using previously published methods [8-12].

## Discussion

To clinically suspect a NOVC infection, it is crucial to update the epidemiological information and obtain a detailed history, including dietary and water exposure. *V. cholerae* is ubiquitous in marine environments and is usually found in estuaries, but it also grows in freshwater and has been isolated from freshwater lakes [1]. Although it is well-known that *Vibrio* species prevalence is higher during the summer season when seawater temperatures rise, recent climate changes such as increased sea surface temperatures due to global warming may further exacerbate the ecological risk of NOVC transmission and infection, particularly in areas with historically low incidence [13]. In developed countries, NOVC infections are often linked to seafood ingestion, particularly raw or undercooked shellfish [14]. In developing countries, however, this pattern is often less clear, due to an increased risk of fecal contamination of food and water sources, as well as cross-contamination of food with seafood [14,15]. In addition to the oral exposure, direct dermal exposure to water, such as seawater, constitutes an alternative route of infection and necessitates consideration. In light of the increasing prevalence of NOVC infections in recent years [2,3], a comprehensive epidemiological risk assessment, encompassing detailed history taking with emphasis on seasonal context, dietary practices, and water exposure, has become imperative.

NOVC is generally considered to be less pathogenic than the cholera toxin-produced O1 and O139 strains, typically causing mild or self-limited gastrointestinal symptoms [2]. However, an increasing number of reports have documented infections of extraintestinal sites such as skin, wound, ear, the biliary or urinary tract, brain, and bacteremia, particularly in patients with underlying immunosuppression [3]. A study from Taiwan found that gastroenteritis was the most common manifestation of NOVC infection (54%), followed by biliary tract infections and primary bacteremia [4]. The incidence of NOVC bacteremia is low, but there has been an observed increase in the number of reported cases worldwide in recent years, particularly in Asia [2]. This condition is more common in males, and over 90% of patients have underlying health issues, most commonly cirrhosis, followed by malignancies and immunosuppressive conditions. The source of infection is identified in 25%-48% of cases, usually related to seafood consumption or exposure to contaminated water. The disease shows seasonal variation, with higher incidence in summer and during monsoon, and lower incidence in winter. Antimicrobials were frequently administered third-generation cephalosporins, fluoroquinolones, and tetracyclines, with a median treatment duration of 14 days [3,5,16]. Mortality rates are relatively high, with reported values reaching up to 39% in patients with bacteremia [4,5]. In this case, the diarrhea had already resolved by the time of admission; however, blood cultures were obtained prior to the administration of antibiotics, which led to an accurate microbiological diagnosis. However, diarrhea was absent in this case, and neither stool nor bile cultures were obtained due to the lack of biliary drainage procedures. We postulate that retrograde cholangitis was triggered by enteritis in the setting of sphincter of Oddi dysfunction following previous biliary interventions. We also considered that multiple risk factors contributed to the development of NOVC bacteremia, including advanced age, male sex, and post-total gastrectomy, as well as poorly controlled diabetes mellitus.

Currently, *V. cholerae* has been classified into more than 200 serotypes based on the O antigen [1]. Toxigenic *V. cholerae* O1 or O139 enter the body through contaminated food or water and subsequently colonize the small intestine. It secretes cholera toxin that is responsible for the activation of adenylate cyclase, which leads to an increase in intracellular cyclic adenosine monophosphate (AMP). This results in the activation of protein kinase A, the inhibition of sodium chloride absorption, and excessive secretion of chloride, bicarbonate, sodium, potassium, and water into the intestinal lumen. The resultant fluid loss can lead to profuse watery diarrhea [1]. Although serotypes other than O1 and O139 have not been correlated with the ability to cause cholera, some reports have implicated cholera toxin-producing strains in the development of cholera-like illness (e.g., serotypes O27, O37, O53, O65) [17].

Despite the rarity of cholera toxin in NOVC strains, these strains often possess numerous other virulence genes that contribute to the pathogenic process. A variety of toxic genes have been identified as contributing to pathogenicity, including the hemolytic element gene (*hlyA*), the protease gene (*hapA*), the cytotoxic actin cross-linking repeats in toxin gene (*rtxA*), the sialidase gene (*nanH*), the heat-resistant toxin (*nag-st*), a type 6 secretion system, and a type 3 secretion system, such as *vscV2 *and *vspD *[18]. The majority of NOVC strains are found to possess *hlyA*, a gene known to contribute to cell detachment and deformation through its interaction with *rtxA*, which is known to elicit a cytotoxic mechanism [19]. Additionally, although few strains carry genes for the type III secretion system, this system has been shown to contribute to bacterial attachment and virulence [20]. The role of these virulence factors in NOVC about pathogenicity remains to be elucidated and is an area that requires further study. In the present case, the strain was found to be negative for *ctx*, a known causative agent of epidemic cholera. However, the strain harbored the El-Tor variant of *hlyA*, *vscV2*, *vspD*, and *rtxA*. Although the expression levels of the virulence genes were not quantified, these factors may contribute to the strain’s ability to cause invasive disease even in the absence of *ctx*. This case supports emerging evidence that the pathogenicity of NOVC strains can be mediated by a diverse repertoire of virulence determinants and emphasizes the need for further genomic and functional studies to better understand the mechanisms underlying severe infections caused by these strains.

## Conclusions

We herein describe a case of acute cholangitis with bacteremia caused by NOVC. Notwithstanding the resolution of diarrhea, it remains imperative to obtain a comprehensive dietary history, with particular attention to the ingestion of raw seafood during the summer season, in addition to performing blood cultures, to facilitate an accurate diagnosis. Additionally, the patient had multiple risk factors, including total gastrectomy, diabetes mellitus with poor glycemic control, and prior biliary reconstruction, suggesting that a previous intestinal infection may have spread beyond the gut and contributed to disease development. Even in cholera toxin-negative strains, the presence of multiple genes encoding alternative virulence factors may also contribute to the development of invasive infections; in cases of NOVC infection, analysis of bacterial virulence genes can provide valuable insights into the clinical course.
